# Recent Advances in the Neuroprotective Properties of Ferulic Acid in Alzheimer’s Disease: A Narrative Review

**DOI:** 10.3390/nu14183709

**Published:** 2022-09-08

**Authors:** Silvia Di Giacomo, Ester Percaccio, Marco Gullì, Adele Romano, Annabella Vitalone, Gabriela Mazzanti, Silvana Gaetani, Antonella Di Sotto

**Affiliations:** Department of Physiology and Pharmacology “V. Erspamer”, Sapienza University of Rome, P.le Aldo Moro 5, 00185 Rome, Italy

**Keywords:** phenolic acid, hydroxycinnamic acids, neuroprotection, neurodegenerative diseases, Alzheimer’s disease, β amyloid, hyperglycemia, type 3 diabetes, clinical trials

## Abstract

Alzheimer’s disease (AD) is a progressive degenerative disorder of the central nervous system, characterized by neuroinflammation, neurotransmitter deficits, and neurodegeneration, which finally leads to neuronal death. Emerging evidence highlighted that hyperglycemia and brain insulin resistance represent risk factors for AD development, thus suggesting the existence of an additional AD form, associated with glucose metabolism impairment, named type 3 diabetes. Owing to the limited pharmacological options, novel strategies, especially dietary approaches based on the consumption of polyphenols, have been addressed to prevent or, at least, slow down AD progression. Among polyphenols, ferulic acid is a hydroxycinnamic acid derivative, widely distributed in nature, especially in cereal bran and fruits, and known to be endowed with many bioactivities, especially antioxidant, anti-inflammatory and antidiabetic, thus suggesting it could be exploited as a possible novel neuroprotective strategy. Considering the importance of ferulic acid as a bioactive molecule and its widespread distribution in foods and medicinal plants, the aim of the present narrative review is to provide an overview on the existing preclinical and clinical evidence about the neuroprotective properties and mechanisms of action of ferulic acid, also focusing on its ability to modulate glucose homeostasis, in order to support a further therapeutic interest for AD and type 3 diabetes.

## 1. Introduction

Alzheimer’s disease (AD) is a progressive degenerative disorder of the central nervous system, characterized by a progressive memory loss, cognitive deficits, and behavior impairments (e.g., poor judgment and capacity to solve problems, difficulties in finding words, reduced visuospatial abilities, personality change) [1]. It is the most common form of dementia, and the reported deaths from AD have increased by more than 145% [2]. Its onset is closely related to the increasingly aging population: it is estimated that its prevalence by 2050 will be doubled in Europe and tripled globally, with more than 1.315 billion AD subjects worldwide [3]. Different AD forms are defined based on the age of onset. In the early-onset form, symptoms start before the age of 65, and in the late-onset form, they manifest later: the latter is the most common form, while the former represents only 5% of the cases [1,4].

AD pathophysiology is very complex, since it is a result of multiple factors, among which the deposition of abnormal β amyloid protein (Aβ) aggregates in the brain (senile plaques) is considered the most well-known etiopathological aspect. Moreover, other contributing factors, including the presence of neurofibrillary tangles containing hyperphosphorylated tau protein within neurons, neuroinflammation, neurotransmitter deficits, and neurodegeneration, which finally lead to neuronal death, have been highlighted [5].

Several epidemiological studies have also shown that mutations in the presenilin proteins (PSEN1; PSEN2) and amyloid precursor protein (APP) are associated with early-onset AD, while those in apolipoprotein E (APOE), especially at e4 allele, increased the risk of late-onset AD [1,6]. Moreover, considering that most AD cases are not hereditary, environmental and genetic factors may act together to finally lead to AD development [7].

To date, the AD etiology is still unclear and the risk factors vary greatly from heart conditions, especially hypertension, mental health, such as mild cognitive impairment (MCI), depression, and anxiety, to drug abuse, including alcohol and smoking [8,9].

Hyperglycemia is emerged as a new risk factor for AD development too; indeed, several epidemiological studies have shown that diabetic patients are two to four times more likely to develop AD [10,11,12]; moreover, people with hyperglycemia are at an increased risk to develop dementia and have a more rapid conversion from MCI to AD [10,11,12]. Accordingly, hyperglycemia has been shown to increase Aβ production and to alter neuronal activity in an animal model of AD, thus exacerbating the pathology [13]. Therefore, disrupting glucose homeostasis seems to play a key role in AD onset.

Glucose homeostasis is strictly regulated by insulin levels [7]: this hormone is responsible for the glucose uptake in neurons, thus stimulating neural growth and survival, suppressing amyloidogenic processing of the amyloid precursor protein (AβPP), and inhibiting the Tau phosphorylation kinase and glycogen synthase kinase 3β [7]. Conversely, insulin resistance in the brain can lead to chronic hyperglycemia, which in turn can deteriorate cognitive function and increase neuroinflammation and neurodegeneration. The presence of brain insulin resistance and insulin deficiency have been reported in both MCI and early-stage AD patients [14]; moreover, insulin and diabetic drugs can improve cognitive function in AD patients [15,16]. Accordingly, along with the deposition of β amyloid aggregates, the “starving brain” [6], due to the neuron inability to process glucose for energetic purposes, could explain cell death in AD.

In line with this evidence, the term Type 3 diabetes has been introduced to describe a form of brain insulin resistance that represents a significant causal factor for AD pathogenesis.

Nowadays, the pharmacological approaches for treating AD are scanty. Usually, anticholinesterases are prescribed at earlier stages, while N-methyl-D-aspartate (NMDA) receptor antagonists are elective treatment strategies [17].

Considering the limited pharmacological options, novel strategies have been addressed to prevent or, at least, slow down the progression of this neurodegenerative disease. Among them, dietary approaches have been proposed to counteract inflammation and oxidative stress, which can contribute to the development of neuroinflammation, mitochondrial dysfunction, impaired neurogenesis, and synaptic plasticity [18]. Particularly, dietary polyphenols have attracted a great attention, due to their pleiotropic biological activities [17,19]. They are chemically characterized by the presence of one or more hydroxyl groups binding to aromatic rings, which are usually linked to sugar moieties and/or other compounds (e.g., amines, carboxylic and organic acids) [17], and can be divided into two major groups, namely flavonoids (i.e., flavonols, flavan-3-ols, anthocyanidins, flavones, flavanones, isoflavones, and chalcones) and non-flavonoids, including phenolic acids, stilbenes, tyrosol, curcuminoids, lignans, saponin, and tannins [20].

Polyphenols are commonly found in edible plants, including fruits, vegetables, olive oil, coffee, and tea, where they exert several protective properties, such as defense against UV radiation and microbial infections [17,20]. They can be also exploited to promote human health, due to their antioxidant power, immune system modulation, endothelial function maintenance and gut microbiota regulation [21]. Moreover, several preclinical studies have highlighted their potential usefulness in preventing or ameliorating cognitive decline [17]. Particularly, it has been demonstrated that natural phenolic acids, especially chlorogenic and caffeic ones, highly abundant in vegetables and fruits, can inhibit the aggregation of proteins involved in the pathogenesis of various neurodegenerative diseases characterized by cognitive deterioration, including AD [22]. This beneficial property seems to be ascribable to their marked antioxidant and anti-inflammatory activities [20].

**Figure 1:**
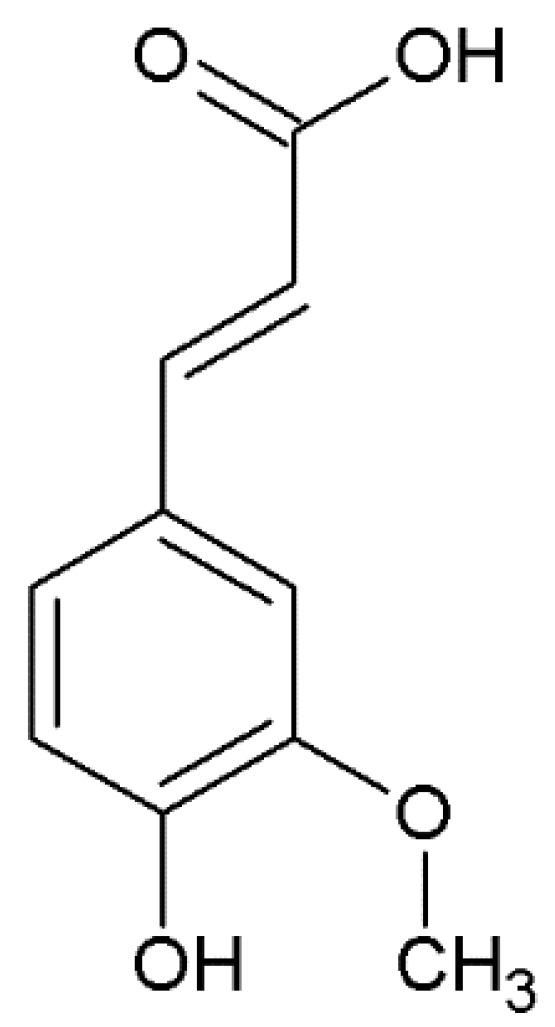
Chemical structure of ferulic acid.

Among phenolic acids, ferulic acid (Figure 1) has recently been proposed as a potential neuroprotective agent. Indeed, it seems to ameliorate AD pathology by preventing neurodegeneration in several brain regions; moreover, it has been shown to inhibit Aβ oligomer aggregations and to exert antioxidant, anti-inflammatory, and anti-apoptotic effects [23].

Considering the importance of ferulic acid as a bioactive molecule and its presence in different foods and medicinal plants, the aim of the present narrative review is to provide an overview of the existing preclinical and clinical evidence about its neuroprotective properties and mechanisms of action, also focusing on its ability to modulate glucose homeostasis, to support a further therapeutic interest for AD and type 3 diabetes. Along with pharmacological evidence, knowledge about the natural occurrence, chemical features, pharmacokinetic properties, and safety profile of ferulic acid have been summarized. Suggestions for future directions are discussed.

## 2. Literature Searching Strategy

The literature has been selected by searching in PubMed and SCOPUS electronic databases, without time limitations and establishing English as preferred language. Moreover, for more specific requirements, Google Scholar and ClinicalTrials.gov were considered. The following searching keywords and their combinations through the Boolean logical operators have been used: “ferulic acid”, “phenolic acids”, “hydroxycinnamic acids”, “natural occurrence”, “neuroprotection”, “neurodegenerative”, “Alzheimer’s disease”, “Alzheimer”, “β amyloid”, “β amyloid aggregation”, “diabetes”, “type 3 diabetes”, “chemical features”, “preclinical studies”, “in vitro”, “in vivo”, “clinical trials”, “antioxidant”, “anti-inflammatory”, “protection”, “apoptosis”, “apoptotic signaling”, “PI3K”, “Akt”, “mTOR”, “Nrf2”, “NF-kB”, “inflammation”, “pharmacokinetic”, and “bioavailability”. The up-to-date studies focused on the beneficial effect of ferulic acid on Alzheimer’s disease and the mechanism of action have been included in the review. Conversely, those regarding the bioactivities of herbal extracts or phytocomplexes containing ferulic acid were excluded, since the true contribution of the substance in combination with other compounds cannot be clearly established.

## 3. Ferulic Acid: General Aspects

### 3.1. Natural Occurrence

Ferulic acid belongs to the phenolic acids, a subfamily of the polyphenol class. Particularly, phenolic acids can be distinguished in hydroxybenzoic acids, whose content in edible plants is generally very low, and hydroxycinnamic acids, which are more common and abundant in fruit, vegetables, cereals, and beverages, such as coffee, tea and wine [20,24].

They occur in plants as free forms or as esters with glucose, quinic acid, tartaric acids, carbohydrates, flavonoids, and as structural components of the cell wall, with both soluble and insoluble hydroxycinnamic derivates [20]. Particularly, soluble hydroxycinnamic derivates are frequently found in fruit and vegetables, while the insoluble derivates are abundant in cereals, where they are frequently associated with the cell wall components such as cellulose and lignin, thus giving structure and rigidity [20,25]. Insoluble hydroxycinnamic acids can be also found on external surface of plants, in form of esters or ethers, in order to produce a layer made up of polymeric waxes [20].

The ubiquitousness of hydroxycinnamic acids is reflected in their significant dietary intake, which can reach, on average, about 211 mg per day, although with differences depending on national and individual dietary habits [26]. It has been estimated that the daily intake of hydroxycinnamic acids ranges from 46.3 to 78.9 mg in children and from 153.6 to 231.8 mg in adults [20]. Cereals (i.e., wheat, maize, rice, barley, sorghum, oats, rye, and millet), represent a significant source of hydroxycinnamic acids [27]. It has been reported that the regular consumption of whole grains is associated with a lower risk of chronic diseases, likely due to the intake of phenolic acids, contained in the bran and germ portions of the grain [25].

Particularly, ferulic acid is contained in fruits (e.g., blueberry, grapes), vegetables (e.g., red beet, radish, pepper, turnip, cucumber, spinach, parsley, tomatoes, carrots) [20,28,29], and, especially, in cereal bran (Table 1) [25]. It is often associated with mono- and disaccharides, glycoproteins, plant cell wall polysaccharides, insoluble carbohydrate polymers, and lignin, and plays an important role in the maintenance of plant structure; indeed, its *trans*-form and esters can cross-link the cell wall, thus contributing to its rigidity [30]; moreover, it is essential to synthesize other secondary metabolites [29].

Cereals represent the major source of ferulic acid, being especially concentrated in the external fraction of grains, although it is available in the whole plant, including roots, stems, leaves, and seeds [27]. The daily intake of ferulic acid through cereals can exceed 100 mg daily for people who habitually consume large amount of these products [26]. It is mainly present in its esterified form with the hemicellulose of the cell walls in wheat; it is present in the whole grains as free, soluble conjugated, and bound forms, usually in the ratio of 0.1:1:100 [25]. It also represents the main phenolic compound of hydroalcoholic wheat extracts, although with differences depending on the crops [27]. Ferulic acid is also the major phenolic acid in corn, sorghum, rye, and rice bran, in which it occurs as a mixture of its esters with triterpene alcohols and sterols, namely γ-oryzanol, which is endowed with antioxidant, anti-inflammatory, and hypocholesterolemic properties [27]. Finally, ferulate derivatives represent the most common phenolic acids of oat [27].

Besides edible plants, ferulic acid is also widely present in medicinal and officinal plants [33], as also shown in Table 2. Particularly, ferulic acid was isolated for the first time from *Ferula assa-foetida* L. [34], a medicinal plant belonging to the Umbelliferae family, cultivated in India, Afghanistan, and Iran [35], and used in Ayurveda and other traditional medicine to relieve gastrointestinal, respiratory and nervous ailments [36]. Other traditional uses of this plant, as an antihelmintic, antispasmodic, antibacterial and antiasthmatic remedy, have been confirmed by pharmacological studies [36]. In two Iranian extracts of *F. assa-foetida*, ferulic acid was found to be the most abundant phenolic, with 4.62 µg/mg and 3.72 µg/mg concentrations in leaf and gum extract, respectively [35].

Ferulic acid is one of the characteristic compounds of the phytocomplex of *Angelica sinensis* (Oliv.) Diels too. This is an endemic plant of China, used in Traditional Chinese Medicine as an antiproliferative and nephroprotective remedy, and to treat menstrual disorders [37]. It is currently used not only in China, but also in Europe and America as a health food product for women’s care [38].

Ferulic acid is mainly contained in *A. sinensis* roots and was found to be one of the major bioactive compounds responsible for its pharmacological properties, being also used as a marker of *A. sinensis* for titration purposes [38]. In a screening of 34 different *A. sinensis* extracts, ferulic acid was found in the concentration range of 0.049–1.502 µg/mL: this huge variability can be ascribed to several factors, such as geographical area, method of cultivation, and harvesting time [38]. Moreover, Dong et al. [39] highlighted that the cultivation of *A. sinensis* at lower temperature gave higher ferulic acid levels, with an increase by about 43% at 15 °C temperature with respect to 22 °C. Ferulic acid is also present in *Levisticum officinale*, a common adulterant of *A. sinensis* [37], and in *Ligusticum wallichii*, whose rhizome is used for its anti-inflammatory, antioxidant, and neuroprotective properties [40].

Species from the Ranunculaceae family, such as *Cimicifuga* spp., widespread in China but also in other temperate regions of the northern hemisphere, such as East Asia, Europe and North America, are also sources of ferulic acid [33,44]. Specifically, the substance has been detected in the aerial parts of *C. foetida,* used as a remedy for sore throat, macula, cold, and headache; its crude extract exerts antiviral, antioxidant, and antiosteoporosis effects. Moreover, ferulic acid derivatives were found in other species of *Cimicifuga* also, such as *C. dahurica* rhizomes, used to treat measles, macula, and gingivitis [33]. Similarly, isoferulic acid was identified in *C. heracleifolia* rhizomes and is considered an index of *Cimicifuga* spp. quality, for which should be not lower than 0.10%.

Ferulic acid, as feruloyl moieties, thus as esters or glycosides, has been also detected in the rhizome of *Sparganium stoloniferum* Buch.-Hamil, a medicinal plant used in Traditional Chinese Medicine to treat menstrual disorders [45], although in different amounts depending on the different geographical areas of cultivation [46].

Finally, plants in the Lamiaceae family have been reported to be a rich source of ferulic acid; indeed, it was detected in the ethanolic extracts of different Croatian medicinal plants, being especially concentrated in those from *Lavandula angustifolia* Miller, *Teucrium* spp. and *Micromeria thymifolia* (Scop.) Fritsch [42]. Moreover, Zgórka et al. [41] reported that *Hyssopus officinalis* L. contained the highest amount of ferulic acid (450 µg/g dry weight), followed by *Lavandula officinalis* Chaix (100 µg/g dry weight), *Rosmarinus officinalis* L. (50 µg/g dry weight)*, Salvia officinalis* L. (50 µg/g dry weight) and *Origanum majorana* L. (50 µg/g dry weight). Conversely, ferulic acid was detected in an extract obtained by subcritical water extraction (8.32 μg/mL) from *Salvia miltiorrhiza* B., but not in a traditional herbal decoction [43].

### 3.2. Chemical Features

Ferulic acid ([E]-3-[4-hydroxy-3-methoxy-phenyl] prop-2-enoic acid) (Figure 1) belongs to the hydroxycinnamic acids, which are characterized by an aromatic ring carrying a carboxy group and one or more hydroxyl groups [27]. Particularly, it possesses a phenolic core with a para-substitution with the unsaturated side chain and it is characterized by an ortho-methoxy group; the side chain, being double-bonded, yields to cis-trans isomerization, leading to the occurrence of ferulic acid as both cis and trans isomers [34].

**Figure 2:**
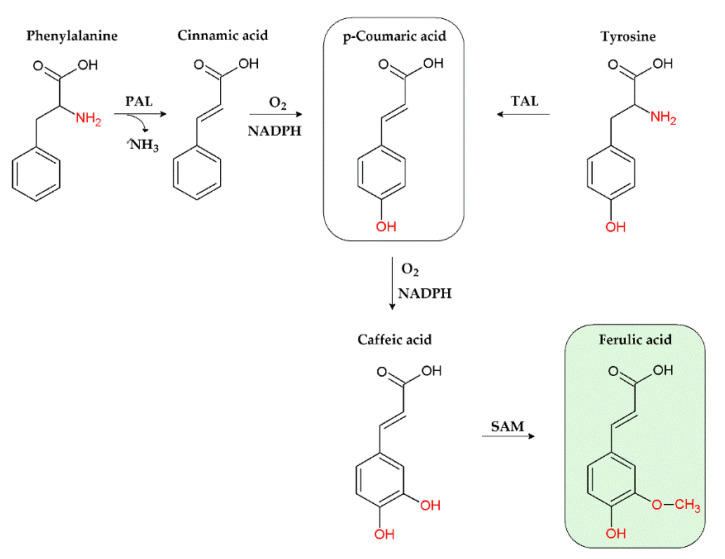
Biosynthesis of ferulic acid through the shikimate pathway. PAL, phenylalanine ammonia-lyase; TAL, tyrosine ammonia-lyase; SAM, S-adenosyl methionine; NADPH, nicotinamide adenine dinucleotide phosphate.

Like hydroxycinnamic acids, ferulic acid is produced by plant cells through the shikimate pathway (Figure 2), which leads to the biosynthesis of both primary and secondary metabolites (e.g., lignins, coumarins, lignans, stilbenes, chalcones, anthocyanins, and flavonoids).

Particularly, its biosynthetic process starts from the deamination of phenylalanine or tyrosine aminoacids, through the intervention of phenylalanine ammonia-lyase (PAL) and tyrosine ammonia-lyase (TAL) enzymes (Figure 2): PAL produces cinnamic acid, which is then converted in p-coumaric acid, while TAL directly generates p-coumaric acid [34]. p-Coumaric acid is then oxidised to caffeic acid, whose O-methylation by S-adenosyl methionine (SAM), leads to ferulic acid [34]. The typical chemical features of ferulic acid confer it several biological properties, which will be discussed in the following sections.

## 4. Pharmacology of Ferulic Acid

### 4.1. Pharmacological Activities and Mechanisms of Action

**Figure 3:**
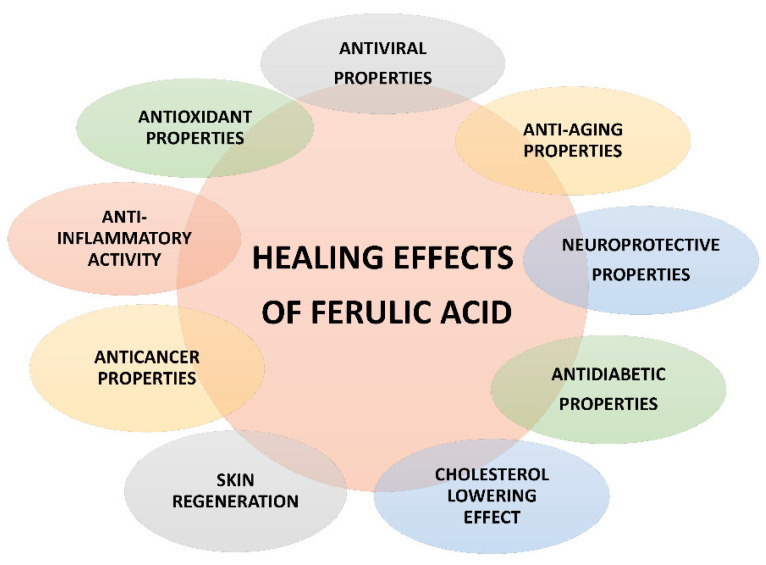
Pharmacological properties of ferulic acid.

Ferulic acid is a multitarget compound, endowed with many bioactivities (Figure 3), thus suggesting it could be widely exploited in the food, pharmaceutical, and cosmetics industries. Particularly, it attracted a great attention for its possible therapeutic applications in different pathologies, including metabolic, cardiovascular and skin disorders, diabetes, hepatotoxicity, viral infections, cancer, and neurodegeneration [23,33,47,48].

Some studies highlighted the ability of ferulic acid and its bound forms, namely γ -oryzanol, to lower the levels and uptake of cholesterol, along with the amount of very low-and low-density lipoproteins, increasing that of high-density lipoproteins [49]. These effects seem to be ascribable to the ability of ferulic acid to inhibit the hydroxymethyl glutaryl CoA reductase and to increase the acid sterol release, thus preventing cholesterol biosynthesis [48]. Furthermore, it was found able to affect glucose metabolism, thus exhibiting antidiabetic effects in various in vitro and in vivo models [50]. It also protected liver from cholestatic and fibrotic injury [51,52], and improved non-alcoholic fatty liver disease (NAFLD) through the modulation of gut microbiota [53].

In addition, it has been shown to be endowed with skin antiaging properties, since it was able to protect fibroblasts, elastin, collagen, and keratinocytes; particularly, it prevented skin damage caused by air pollution and UV radiation through radical scavenging mechanisms [54,55]. Its antiblemish and skin lightening properties, likely due to the inhibition of melanocyte proliferation and tyrosinase enzyme, have been highlighted also [54,55].

Other studies also reported the regenerative and angiogenic properties of ferulic acid [23], along with antiviral and anti-inflammatory effects against influenza A and respiratory syncytial viruses [47]. Recently, it was found able to inhibit SARS-CoV-2 membrane protein in a high-end molecular docking analysis [56].

Further evidence highlighted the ability of ferulic acid to prevent oxidative stress and to promote the activity of cytoprotective enzymes in lung, gastrointestinal, and breast cancers both in vitro and in vivo, thus suggesting a potential interest as an anticarcinogenic agent; finally, it boosted the immune system, thus reducing the side effects of radio- and chemotherapy [48]. Promising protective properties of ferulic acid were also displayed in different models of neuronal damage, which were mainly ascribed to its anti-inflammatory and antioxidant properties [57]. Details about the anti-inflammatory and antioxidant mechanisms of ferulic acid involved will be discussed in the following paragraphs.

#### 4.1.1. Anti-Inflammatory Properties

Inflammation has been associated with several age-related and chronic diseases, such as AD and diabetes [58]. The term “inflammaging” has been recently coined to point out a body state characterized by a chronic low-grade inflammation and persistent secretion of proinflammatory cytokines by inflammatory cells (e.g., neutrophils, monocytes, macrophages), which can modify cellular function; during inflammaging, C-reactive protein (CRP) levels are also increased, and both innate and adaptive immunity are impaired, so leading to an inadequate anti-inflammatory response [58]. Therefore, the persistence of this inflammatory state further worsens clinical outcomes.

Several studies have highlighted the anti-inflammatory effects of ferulic acid in a variety of diseases, such as AD, diabetes, and liver and kidney dysfunctions [33,59]. It seems able to fight inflammation mainly by inhibiting the secretion and expression of related inflammatory factors through affecting multiple molecular pathways [33]. Particularly, ferulic acid has been shown able to reduce the neuroinflammation induced by chronic unpredictable mild stress in the prefrontal cortex through the inhibition of the nuclear factor kappa-B (NF-κ B), a key mediator of proinflammatory cytokine signaling pathway, which promotes the synthesis of interleukin (IL)-1β, IL-6, and tumor necrosis factor alpha (TNF-α), leading to neuroinflammation [60]. In the same experimental conditions, it also inhibited the NLR pyrin domain-containing protein 3 (NLRP3) inflammasome [60].

A down-regulation by ferulic acid of proinflammatory molecules, such as nitric oxide synthase (iNOS), cyclooxygenase-2 (COX-2), TNF-α, IL-1β, vascular cell adhesion molecule-1 (VCAM-1), and intercellular adhesion molecule-1 (ICAM-1), has been observed following NF-κB inhibition [61,62]. Particularly, VCAM-1 and ICAM-1 are highly involved in the immune response of inflammatory reaction, since they promote the migration and infiltration of inflammatory cells, especially monocytes, thus exacerbating the inflammatory response [33]. Therefore, modulating their expression by ferulic acid could be an interesting strategy to reduce inflammation. In this respect, ferulic acid (100 mg/kg, i.v.) inhibited the expression of ICAM-1 and macrophage-1 antigen (Mac-1) mRNA in ischemic striatum and reduced macrophage infiltration, thus leading to a downregulating of inflammation [63]. Similarly, the pretreatment of human umbilical vein endothelial cells (HUVEC) with ferulic acid before gamma ray irradiation determined a reduction of both ICAM-1 and VCAM-1 expression [64].

Ferulic acid was also able to affect the mitogen activated protein kinases (MAPKs) pathway, by inhibiting the phosphorylation of MAPKs, including p38 and c-Jun N-terminal kinase (JNK), in LPS induced inflammatory response of bovine endometrial epithelial cells (BEECs): this led to a reduction of proinflammatory cytokines (IL-1β, IL-6, TNF-α and IL-8) mRNA expression [65]. Moreover, ferulic acid reduces the liver damage induced by acetaminophen in a mouse model of hepatotoxicity by inhibiting the expression of toll like receptor 4 (TLR4), which is involved in the phosphorylation of p38 MAPK and IκB; as a consequence, a reduced release of proinflammatory mediators, namely IL-1β and NF-κB, was found [66].

Another target through which ferulic acid can impair inflammatory response is the peroxisome proliferator activated receptor γ (PPARγ); indeed, its activation in monocyte macrophages determines a downregulation of several inflammatory factors, such as IL-1β, IL-6, TNF-α, iNOS and COX-2 [33]. In a rat nephrotoxicity model induced by gentamicin, the administration of 100 mg/kg ferulic acid determined anti-inflammatory and renal protective effects by enhancing the CAT activity and PPAR γ gene expression [67]. Similar results were obtained in a methotrexate-induced nephrotoxicity model, in which ferulic acid upregulated PPARγ and Nrf2 expression in renal cells, and inhibited the NF-κB/NLRP3 inflammasome axis and apoptosis, through PPARγ activation [68].

Overall, present evidence highlights that the anti-inflammatory effects of ferulic acid are closely related to a modulation of NF-κ B and p38 MAPK signaling pathways, along with a down-regulation of CAM, and an activation of PPARγ.

#### 4.1.2. Antioxidant Properties

Nowadays, there is wide recognition of the key role of oxidative stress in the onset and exacerbation of several pathologies, among which neurodegenerative diseases and metabolic ailments [69]. Usually, oxidative stress can be counteracted in the human body by both enzymatic (e.g., superoxide dismutase, catalase, glutathione peroxidase) and non-enzymatic (e.g., vitamin C, vitamin E, glutathione, α—lipoic acid) antioxidant systems; however, under persistent oxidative imbalance, marked levels of oxidative species can determine the development of a low grade chronic inflammatory state, so leading to tissue degeneration, premature aging, and apoptosis [69]. Consequently, fighting oxidative stress appears as a promising strategy to prevent the development of non-communicable diseases.

Several studies have highlighted the antioxidant properties of ferulic acid, mainly ascribed to its peculiar chemical features; indeed, it was able to scavenge free radicals, inhibit the generation of reactive oxygen species (ROS) and modulate several signalling pathways involved in oxidative stress control [33].

Regarding the radical scavenging activity, ferulic acid can transfer a hydrogen to radical species thanks to the presence of the hydroxy groups, leading to the production of a phenoxy radical, which is further stabilized by the ethylenic unsaturated side chain, thus leading to the termination of the free radical chain reaction [70]. Furthermore, the *ortho*-methoxy group helps stabilize the phenoxy radical through electron delocalization [71]. Finally, it has been reported that ferulic acid esters possess improved radical scavenger activities [70]. The ability of ferulic acid to form stable phenoxy radicals, by acting as a hydrogen donor, confers it also the ability to protect lipid membrane from autoxidation [23].

Ferulic acid may also inhibit the generation of reactive oxygen species (ROS) through the Fenton reaction, acting as a chelator of metals (i.e., Fe and Cu), which take part in it [23,72]. Moreover, it showed to modulate enzymes involved in free radical production and antioxidant defenses [33]. Alim et al. [73] found that ferulic acid inhibited aldose reductase (AR) enzyme, which is involved in the reduction of aldehydes or ketones to the corresponding alcohols by using nicotinamide adenine dinucleotide phosphate (NADPH) as coenzyme. Lowered NADPH levels are closely related to oxidative stress; therefore, inhibiting AR by ferulic acid can prevent the consumption of NADPH and, consequently, the redox status impairment [73]. Acting as an AR inhibitor, ferulic acid seems also to improve diabetes-associated hypertension, by restoring NO (nitric oxide) production, reducing leukocyte infiltration, inhibiting endothelial cell pyknosis and decreasing ROS formation [74].

It has been also shown that the pretreatment of HEK (human embryonic kidney) 293 cells with ferulic acid reduced the H_2_O_2_-induced ROS levels by increasing the activity of the antioxidant superoxide dismutase (SOD) and catalase (CAT) enzymes [75]. Accordingly, Chowdhury et al. [76] showed that the administration of ferulic acid (50 mg/kg) in a diabetic rat model determined a reduction of intracellular ROS level, through the modulation of SOD and CAT enzymes; a lowering in the levels of malondialdehyde (MDA), a lipid peroxidation marker, and advanced glycation end products (AGEs), and in the activity of xanthine oxidase (XO), both involved in ROS formation, were observed too.

The antioxidant activity of ferulic acid has been found associated to the modulation of several signaling pathways, and to an increased expression of the nuclear translocation of the transcription factor NF-E2-related factor (Nrf2) [77]. Particularly, Nrf2 binds the antioxidant responsive element (ARE) in the promoter region of the heme oxygenase-1 (HO-1) gene, and increases its transcription, thus potentiating the endogenous antioxidant defenses [77]. Ferulic acid has been found able to upregulate HO-1, thus increasing the production of bilirubin, which acts as an efficient ROS scavenger, in human umbilical vein endothelial cells (HUVEC) under radiation-induced oxidative stress [77].

It was also highlighted that the activation of extracellular signal-regulated kinase (ERK) and phosphatidylinositol 3-kinases/protein kinase B (PI3K/AKT) signaling pathways plays an important role in the modulation of the Nrf2 cascade; indeed, their inhibition by a specific antagonist determined an attenuation of the Nrf2-induced HO-1 gene expression [59]. Along with the HO-1 upregulation, an increased expression of other antioxidant genes, such as glutamate-cysteine ligase catalytic subunit (GCLC), glutamate-cysteine ligase regulatory subunit (GCLM), and NADPH quinone oxidoreductase-1 (NQO1) were induced by ferulic acid [77]. Therefore, it seems that the induction and activation of Nrf2, through PI3K and ERK signaling pathways, plays a key role in the cytoprotective effect of ferulic acid against oxidative stress.

### 4.2. Pharmacokinetic Properties

**Figure 4:**
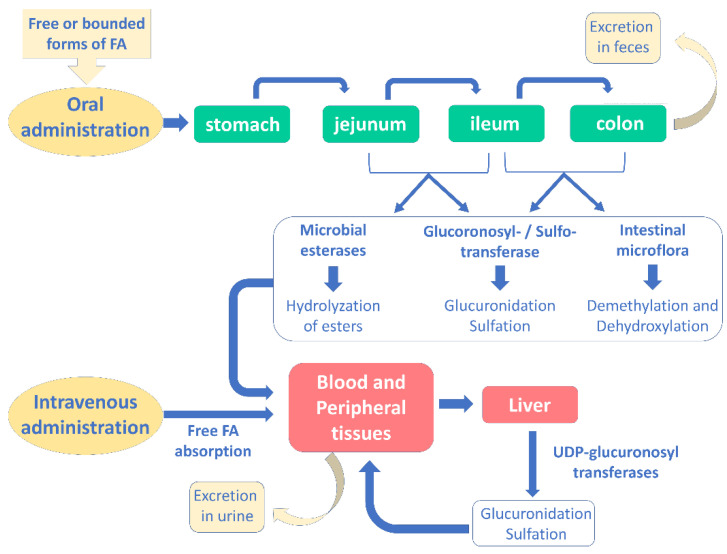
Absorption, metabolism and excretion of ferulic acid in human body after oral and intravenous administration (modified from [30]).

Several studies have investigated the pharmacokinetic profile of ferulic acid and its bounded forms after oral administration, while no studies have been performed after intravenous administration. As displayed in Figure 4, after ingestion, ferulic acid is absorbed in several gastrointestinal portions, namely in the stomach, small intestine, jejunum, ileum, and colon [30].

Free ferulic acid is usually absorbed before reaching the ileum; conversely, when it is bound to fibers, it must be degraded in the hindgut before absorption [78]. Indeed, colon is the most important site for the absorption of ferulic acid: here, microbial esterases hydrolyze its esterified forms to release free ferulic acid, which can be retrieved in plasma [78]. Consequently, after hydrolysis the esterified forms seem to follow the same metabolic fate of the free ferulic acid.

Passive transcellular diffusion and facilitated transport are responsible for ferulic acid absorption [30]. The first mechanism is mainly involved in gastric absorption, being highly dependent on the environmental pH, pKa and compound hydrophobicity, while ferulic acid possesses a pKa equal to 4; therefore, it can be passively transported in an undissociated form in the low gastric pH environment [30]; conversely, a facilitated transport is required in the intestinal absorption, where the environment pH is >5 [78]. Regarding the involved transporters, several hypotheses have been postulated: some studies have shown the potential involvement of a Na^+^-dependent transport mechanism, while others of a H^+^-dependent carrier [79]. Moreover, ferulic acid seems to be taken up by monocarboxylic acid transporters, which are involved in gastric absorption [80].

Metabolism of the substance mainly takes place in the liver, where the major metabolites are represented by ferulic acid sulfoglucuronide, glucuronide, and sulfate [78]. Sulfotransferases and UDP glucuronosyl transferases are responsible for the conjugation reactions, although intestinal mucosa and kidney may take part in this process. Other metabolites of ferulic acid include m-hydrophenylpropionic acid, feruloylglycine, dihydroferulic acid, vanillic acid, vaniloylglycine, feruloyl-sulfate and trans-feruloyl-4-O-b-D-glucuronide, which are obtained by double bond reduction, demethylation, C3 or C4 dehydroxylation, as well as methylation [30]. Usually, ferulic acid metabolites are effluxed in a transporter(s)-dependent way [81].

Its highest plasma concentration varies greatly depending on the investigated species: it is reached at 24 min and 2 min after ingestion in humans and rats, respectively [81,82]. Moreover, the half-lives for distribution and elimination have been reported at 10 and 60–106 min, respectively [82]. Chang et al. [83] have shown that 30 min after ferulic acid administration (521 μmol/kg) in rats, it was mainly found in kidney (82 μg/g), lung (34 μg/g), liver (28 μg/g), spleen (22 μg/g), uterus (15 μg/g), heart (14 μg/g), and brain (2.6 μg/g). Other studies reported that 30 min after its oral administration, ferulic acid was recovered in plasma, mainly as glucuronidated and sulfoglucuronidated forms (76%), and at lower level in its free form (24%) [84].

Regarding excretion, urine is the favorite way: about 5% of free ferulic acid and a total 11–25% of free and bounded forms were recovered in urine; conversely, only the 0.5–0.8% of ingested ferulic acid has been found in rat feces, thus indicating a very efficient absorption [30]. However, it should be outlined that ferulic acid bioavailability greatly depends on the food matrix, although conflicting results exist; indeed, encapsulated ferulic acid was found to possess a bioavailability 4-fold higher than that of the free form [85]; moreover, other studies displayed an improved absorption of the ferulic acid in its conjugated forms [86,87]. It is likely that ferulic acid bioavailability is affected by the complexity of the polysaccharide matrix, leading to different results [30].

Finally, it is noteworthy that ferulic acid esterified forms have been shown to act as a prebiotic, since they stimulate the growth of eubacteria, such as *Lactobacilli* and *Bifidobacteria*, in the human gastrointestinal tract, so preserving the homeostasis of gut microbiota, whose impairment can contribute to several disease, including neurodegeneration [30].

## 5. Ferulic Acid and Alzheimer’s Disease (AD)

AD is a chronic neurodegenerative disorder which progressively leads to the impairment of cognitive and physical functions [1]. The aggregation and deposition of Aβ_36–43_ peptides in the form of oligomers or fibrils in the synaptic space trigger the activation of several kinases, namely glycogen synthase kinase-3 beta (GSK3b), cyclin-dependent kinase 5 (cdk5), and dual-specificity tyrosine phosphorylation-regulated kinase 1A (DYRK1A) which, in turn, are responsible for tau hyperphosphorylation and aggregation in neurofibrillary tangles. As a result, an impairment of mitochondrial activity occurs in the neuron; moreover, increased ROS production, decreased antioxidant enzyme activities, and the inflammatory response activation by microglia cells can be observed [88]. Noteworthy, also, the apoptosis cascade is triggered, so leading to the injury of cholinergic areas involved in cognitive performance, such as amygdala, hippocampus and cortex [59].

Considering the AD pathophysiological framework, ferulic acid has been approached as a possible neuroprotective strategy against AD due to its promising antioxidant and anti-inflammatory properties. A comprehensive overview of the in vitro, in vivo and clinical studies focused on the potential healing effects of ferulic acid in AD is reported in the following paragraphs.

### 5.1. In Vitro Studies

Several studies have been performed to investigate the potential role of ferulic acid against AD by exploiting cell models. In particular, Kikugawa et al. [89] showed that the pretreatment of primary cerebral cortical neurons with ferulic acid exerted a protective effect towards the Aβ_25–35_ induced cytotoxicity; moreover, ferulic acid was able to inhibit the aggregation of Aβ_25–35_, Aβ_1–40_, and Aβ_1–42_ and to destabilize pre-aggregated Aβ. The neuroprotective properties of ferulic acid were also observed in microglial cells stimulated with LPS; indeed, an inhibition of TNF-α, IL-6, IL-1, and NO release and a reduction of COX-2 and iNOS activity were observed [90]. Similarly, in Neuro-2a cells triggered with H_2_O_2_, a down-regulation of iNOS, eNOS, COX-2, IL-1β, caspase-9, and BCL-2 genes, and an up-regulation of brain-derived neurotrophic factor (BDNF) gene were observed after treatment with ferulic acid [91].

Protective properties were also highlighted in the pheochromocytoma PC12 cell line, which is commonly used for neuroprotective investigations: ferulic acid was found able to inhibit the production of TNF-α and IL-1β induced by LPS and the up-regulation of phosphodiesterase 4 (PDE4) activity; moreover, a down-regulation of cAMP-response element binding protein (CREB) and pCREB induced by LPS was observed [92]. In the same in vitro model, it has been also highlighted that ferulic acid increased the viability of hypoxia stressed PC12 cells: this effect was ascribable to its antioxidant power [93]. Indeed, it prevented membrane damage, scavenged free radicals, increased SOD activity, and decreased the intracellular free Ca^2+^ levels, lipid peroxidation, and the release of prostaglandin E2 (PGE2); also, a reduction of p-p38 MAPK, caspase-3, and COX-2 activation was observed, thus meaning that ferulic acid also exerted antiapoptotic and anti-inflammatory effects [93]. Finally, Rosini et al. [94] tested a derivative of ferulic acid, in which the phenolic acid was conjugated with the NMDA agonist memantine, in order to explore the possible connection between NMDA receptors, oxidative stress and amyloid-β peptide in AD. The ferulic acid conjugate was able to protect the human SH-SY5Y cells from Aβ neurotoxicity and to reduce the ROS-induced neuronal death [94].

### 5.2. In Vivo Studies

The potential usefulness of ferulic acid in AD has been also investigated in different rat and mice models, among which the APP/PS1 transgenic mice and the intraventricular injection of Aβ were the most used [3].

Yan et al. [95] reported that IL-1β production, neuroinflammation, and gliosis, induced by the intracerebroventricular (i.c.v.) injection of Aβ in the mouse hippocampus, were counteracted by the pre-treatment with ferulic acid (14–19 mg/kg/day per os) for 4 weeks; moreover, the phenolic acid was able to improve memory loss. Similarly, another study showed that a long-term (4-week) pretreatment with ferulic acid (0.006% *w/v* in drinking water) in mice suppressed the microglia activation and interferon-gamma (IFN-γ) release in the hippocampus at 8 h after the intracerebroventricular injection of Aβ_1–42_ [96]. The same authors also showed that ferulic acid prevented the Aβ_1–42_-induced increase of endothelial nitric oxide synthase (eNOS) and 3-nitrotyrosine (3-NT) and suppressed the IL-1 α immunoreactivity in the hippocampus [97].

Furthermore, Jin et al. [98] observed a strong reduction of IL-1β and an increased phosphorylation of both ERK and Akt in rat hippocampus after ferulic acid administration (50–250 mg/kg/day orally for 4 weeks), which resulted in lowered inflammation and increased pro-survival signaling. In addition, the inhibition of apoptotic cascade, particularly caspase-9, -3, and -7 activation induced by Aβ_1–40_ (i.c.v. route), was reported after pre-treatment with ferulic acid (100 and 200 mg/kg intragastrically for 3 weeks) [99].

Kim et al. [100] highlighted that ferulic administration (0.002–0.005% in drinking water) for 28 days improved the trimethyltin-induced cognitive deficit: an increase in the choline acetyltransferase activity was hypothesized as a possible mechanism of action. Similarly, Mamiya et al. [101] demonstrated the neuroprotective and nootropic effects of ferulic acid after a short-term administration by a parenteral route (5 mg/kg subcutaneously for 6 days) in mice deprived of reduced glutathione. The treatment counteracted oxidative stress induced by deprivation and significantly improved the mouse cognitive abilities.

Furthermore, a dietary supplementation with sodium ferulate (SF, 100–200 mg/kg body weight for 4 weeks) in 21-month-old rats was able to counteract the increased release of pro-inflammatory cytokines induced by age [102]. Indeed, a reduction of IL-1β in the rat hippocampus, along with a modulation of both ERK1/2 and Akt, which possess a marked neuroprotective activity, were observed [102]. Hamaguchi et al. [103] showed that administering ferulic acid (0.5% in food) for 10 months reduced Aβ deposits in an AD transgenic mouse (Tg2576) model, while Beibei et al. [104] reported an improvement in the mouse learning and cognitive skills along with a reduction of GFAP (glial fibrillary acidic protein) expression in the hippocampal CA1 region.

A reduction of neuroinflammation, associated with a reduced Aβ deposition and IL-1β levels in the frontal cortex and an enhancement of cognitive performance, was also highlighted in the transgenic APP/PS1 mouse model after oral administration of ferulic acid for 6 months [105]. In the same AD model, Mori et al. [106] found that ferulic acid (30 mg/kg orally for 6 months) decreased Aβ production from APP and neuroinflammation, and stabilized oxidative stress; consequently, cognitive tasks were improved. Similarly, cognitive function and memory were found improved after administration of ferulic acid (20–100 mg/kg intragastrically for 2–4 weeks) in an AD model induced by i.c.v. injection of Aβ_1–40_: the effect was correlated to the antioxidant and anti-inflammatory properties of ferulic acid [107]. Accordingly, improved cognitive functions and protective effects on neuron survival by ferulic acid (0.1 µmol/g/day orally for 42 days) were reported in a murine AD model induced by i.c.v. injection of Aβ_25–35_ [89].

Another study showed that ferulic acid, administered intragastrically (30 mg/kg) for 3 months, improved memory in the transgenic APP/PS1 mice, and reduced Aβ deposits, astrocytosis, microgliosis, synaptotoxicity, amyloidogenic APP processing, neuroinflammation and oxidative stress [108]. Similar results were also highlighted by other authors, as reported in Table 3 [109,110,111,112,113]. Along with the amelioration of Aβ plaque deposition, Wang et al. [114] recently found that ferulic acid prevented the reduction of density and diameter of hippocampal capillaries, thus favoring the oxygen and nutrients supply and removal of metabolic wastes from the brain, which finally led to improved spatial memory.

### 5.3. Clinical Trials

Scanty clinical trials evaluating the potential therapeutic efficacy of ferulic acid in dementia are available; however, the available studies focused on combinations of the phenolic acid and other agent, while none tested this substance alone. Particularly, our literature searching allowed us to retrieve only 3 studies, which were designed to evaluate the beneficial effects of a Feru-guard^®^ 100M formulation on mild cognitive impairment. Feru-guard^®^ is a dietary supplement, containing a mixture of *Angelica archangelica* and ferulic acid in the ratio 1:5, claimed to improve the behavioral and psychological symptoms of dementia [115].

Kimura et al. [116] investigated the efficacy of Feru-guard^®^ in the treatment of behavioral and psychological symptoms in frontotemporal lobar degeneration and dementia with Lewy bodies. A 4-week prospective, open-label trial, in which patients (n = 20) assumed daily Feru-guard^®^ (3.0 g/day), was designed. Behavioral and psychological symptoms of dementia were assessed at baseline and 4 weeks after the start of treatment, using the Neuropsychiatric Inventory, which is a validated instrument for evaluating psychopathology in dementia. The obtained results highlighted a decrease in the Neuropsychiatric Inventory score in 19 out of 20 patients and in the overall score. The treatment also led to a significant amelioration of behavioral and psychiatric symptoms of dementia such as delusions, hallucinations, aggression, and anxiety. Based on these results, the authors concluded that the dietary supplement could be effective for treating the behavioral and psychological symptoms of dementia in frontotemporal lobar degeneration and dementia with Lewy bodies.

Matsuyama et al. [117] carried out an open-label, interventional multi-institutional joint study between October 2014 and August 2018, in which patients diagnosed with MCI at Kobe University Hospital, based on the Alzheimer’s Disease Neuroimaging Initiative criteria for MCI, were enrolled; furthermore, the presence of largely intact general cognition and functional performance and the absence of dementia diagnosis were needed [118]. Patients were divided in two groups: the intervention group (n = 10), which used one pack (1.5 g) Feru-guard^®^ 100M before both breakfast and dinner every day for 48 weeks, and the control group (n = 7), which did not use the supplement. One pack of Feru-guard^®^ 100M includes 100 mg ferulic acid and 20 mg *A. archangelica* extract. Differences between the two groups were assessed by examining changes in Aβ deposition, brain volume, and cognitive function at regular intervals; however, after 48 weeks, no significant differences were highlighted between groups.

Kudoh et al. [115] conducted a multicenter, randomized, double-blind, placebo-controlled prospective trial examining the Feru-guard^®^ efficacy in MCI. Participants aged 65 to 85 years old with MCI were enrolled and randomly allocated in the active group (n = 30), receiving a Feru-guard^®^ daily dose equal to 200 mg of ferulic acid and 40 mg of *A. archangelica* extract, or in the placebo group (n = 26). Participants took the supplement or placebo before breakfast and dinner every day throughout the trial period (48 weeks). In the intention-to-treat population, Mini-Mental State Examination (MMSE) scores were significantly better at 24 weeks in the active group and in the per protocol population. Similarly, significant differences were seen for the mixed effect models for repeated measures (MMRM) and for the Alzheimer’s Disease Assessment Scale-Cognitive Subscale, Japanese version (ADAS-Jcog) at 24 and 48 weeks. Based on that evidence, authors stated that Feru-guard^®^ is clinically effective on cognitive functioning among older adult with MCI.

## 6. Ferulic Acid as a Possible Strategy to Fight Type 3 Diabetes

As previously stated, hyperglycemia has recently emerged as a risk factor for the development of AD. Indeed, some studies have highlighted that diabetes patients are more likely to develop AD [10,11,12]. Several pathological features are shared between these two diseases, among which the release of proinflammatory mediators by NF-κB activation, the advance glycation end-product (AGE) formation, the neuronal membrane lipid peroxidation, and alteration in the acetylcholinesterase (AChE) and butyrylcholinesterase (BChE) levels [119]. This general dysfunctional pattern can determine an exacerbation of Aβ production, oxidative stress, mitochondrial dysfunction, and neurofibrillary tangle formation, so leading to loss of neuronal functions and development of AD during a diabetic condition [119]. For this reason, the term Type 3 diabetes has been coined to point out a neuroendocrine disorder characterized by the development of brain insulin resistance and seems to represent the progression of type 2 diabetes mellitus (T2DM) to AD [120].

Therefore, at present, the scientific research has been addressed towards the study of existing drugs and/or bioactive compounds which can be exploited to counteract this new emerging disease [15,16]. For example, the antidiabetic drugs insulin and pioglitazone have already shown promising results [15,16]. However, natural compounds, especially polyphenols, have also been investigated due to their multitargeted properties and common supplementation by diet [121]. Among them, ferulic acid seems to be an attractive compound since it is endowed with both neuroprotective and antidiabetic properties [50,57].

Particularly, it was found able to inhibit α-glucosidase enzyme, so lowering glucose absorption and postprandial hyperglycemia [122]. Moreover, in alloxan- and streptozotocin-induced diabetic models, it displayed beneficial effects by exhibiting antioxidant features and preventing inflammation in the pancreas, liver, kidney and serum [59]. Ferulic acid was also able to control blood glucose levels by preserving β cells viability and, thus the insulin production in the pancreas, so decreasing toxicity and oxidative stress; wound healing and regenerative properties in diabetic rats were also reported [123].

Despite the evidence presented in the literature about the neuroprotective and antidiabetic properties of ferulic acid, only one recent study by Park et al. [124] has investigated its therapeutic potential in type 3 diabetes. The authors [124] evaluated the effects of ferulic acid on brain and systemic insulin resistance, and on memory function, by using non-obese type 2 diabetic animals with memory deficits. The animal model was obtained by partial pancreatectomy and Aβ_25–35_ infusion into the rat hippocampus. Ferulic acid was orally administered through a high-fat diet, to exacerbate insulin resistance, at the dose of 50 mg/kg body weight/daily for 49 days, while in AD (AD-CON) and normal (Normal-CON) control groups an equivalent dose of dextrin was used.

The ferulic acid treatment determined a lowered body weight gain with respect to the controls, and hindered the memory impairment through the inhibition of amyloid-β deposition and neuroinflammation compared to the AD-CON.

Regarding glucose metabolism, ferulic acid decreased the serum insulin concentrations and improved glucose intolerance; furthermore, an increase of brain derived neurotrophic factor (BDNF) and ciliary neurotrophic factor (CNTF) mRNA expression was observed in the hippocampus of the ferulic acid group. Finally, an increase of Akt and GSK-3β phosphorylation was highlighted with respect to AD-CON, suggesting a reduction of brain insulin resistance.

Overall, the study by Park et al. [124] highlighted that ferulic acid supplementation by diet could represent a promising strategy to improve memory function by increasing insulin sensitivity at both brain and systemic level, likely by a reduction of oxidative stress and inflammation, which are common pathological mechanisms of AD and diabetes. Therefore, preserving insulin sensitivity by ferulic acid can be helpful to prevent or at least delay memory dysfunction.

However, it must be outlined that some limitations are present in the study as stated by the same authors. For example, the animal model used for memory deficit (i.e., hippocampal amyloid-β infusion) is not representative of the true etiology of human AD. Moreover, the content of ferulic acid in blood and hippocampus was not measured: this is an important issue to consider, since ferulic acid is characterized by a low lipophilicity (cLog P ~1.5), which implicates a poor blood-brain barrier (BBB) permeability and aqueous solubility, which are the major limits associated with ferulic acid as a druggable agent for AD [125].

In support, previous studies have shown that by administering 521 μmol/kg ferulic acid in the rat, only 2.6 μg/g reached the brain [83]. Moreover, after oral administration, ferulic acid is rapidly conjugated and is present in plasma mainly as glucuronidated and sulfoglucuronidated forms (76%) [84].

In order to improve ferulic acid bioavailability, several novel compounds have been designed by using ferulic acid as a template; indeed, the presence of acid and phenolic functional groups in its chemical structure provides the needed space for multiple structural modifications in order to obtain druggable molecules with a more balanced hydrophilicity/lipophilicity ratio retaining its inherent properties [125]. Furthermore, lipid-based formulations, such as liposomes, have been prepared as a new delivery system to allow a better absorption of ferulic acid [59].

Finally, we should not forget the role played by gut microbiota in the release of ferulic acid from dietary sources, permitting absorption. Therefore, a complete and healthy microbiota is necessary to support the action of ferulic acid, which is considered a key target for mediating communication between the commensal microbiota and the brain [126]. However, alterations in the diversity or structure of gut microbiota, known as dysbiosis, have been reported in both AD and diabetes [127]. Consequently, a lower amount of this compound could be released during these pathological conditions, thus likely limiting its healing effects. This evidence suggests the need of further deeper studies, focusing not only on the role of ferulic acid in type 3 diabetes but also on the pharmacokinetic features required to exploit its beneficial properties.

## 7. Safety Profile

Few studies have investigated the tolerability profile of ferulic acid. An in vitro study showed that it was not cytotoxic in NIH-3T3 fibroblasts and 3T3-L1 adipocytes, while early signs of toxicity were observed in HepG2 liver cancer cells at the concentration of 500 μg/mL. Similarly, ferulic acid did not affect the viability of platelets, leukocytes, and erythrocytes up to the concentration of 300 μg/mL [128]. Conversely, it produced cytotoxic effects in mouse fibroblasts (L929) at the concentration of 40 μg/mL and in human monocytes (U937) and colon cancer cells (Caco2) at the same concentration [129]. Furthermore, 20 μg/mL ferulic acid reduced the occludin expression in an intestinal tridimensional model, although without affecting the structure of the epithelial layer [129]. Present results allow us to hypothesize that ferulic acid could have different toxic effects depending on the specific cell lines; therefore, it should be used with caution [129]. Despite the previous evidence, Salau et al. [130] did not find signs of toxicity of ferulic acid in hippocampal neuronal cell lines HT22 cells, thus concluding that the substance seems to be safe in healthy brain cells. These results were further corroborated by the predicted oral LD_50_ value which was equal to 1772 mg/kg, so allowing classification of ferulic acid in a toxicity class of 4, meaning that it is safe when orally consumed [130]. In support, a previous study by Tada et al. [131] found that ferulic acid has a low degree of toxicity after oral administration, the LD_50_ being equal to 2445 mg/kg and 2113 mg/kg in male and female rats, respectively, and to 3200 mg/kg in mice [59]. Furthermore, Xu et al. reported a LD_50_ equal to 866 ± 28 mg/kg after intravenous administration of ferulic acid in mice; the main toxicity signs of ferulic acid were spasticity, tremor, hind limb ankylosis, and death, which occurred within 6 h and were reduced in combination with the natural compound ligustrazine [132]. Finally, in a rat subchronic toxicity study, 0.6 g/kg/day ferulic acid, administered intragastrically for 3 months, did not produce hematological or pathophysiological changes [133].

Possible side effects of ferulic acid treatment with quercetin, including increased incidence of cataract, injured glomerules, and renal cell carcinoma, were highlighted in a streptozotocin (STZ)-induced DM rat model; however, considering that STZ alone was able to induce renal carcinoma, the potential tumorigenicity of ferulic acid requires to be better investigated. Another in vivo study showed that ferulic acid (70 mg/kg) induced renal damage in a chronic kidney disease model induced by doxorubicin, after long-term treatment [134]. Finally, ferulic acid has been reported to reduce the activity of cytochrome P450 1A (CYP1A) and increase UDP-glucuronosyltransferase (UGT) activity, although without clinical evidence about its possible interference with bioavailability of co-administered drugs [59].

Based on the present evidence, although several findings seem to highlight a tolerability of the substance, others suggest possible concerns which require further studies to clarify the real safety profile of ferulic acid, in order to better exploit its healing properties for therapeutic purposes.

## 8. Conclusions and Future Directions

There is a growing scientific interest in unraveling the benefits of ferulic acid in neurodegenerative diseases, especially AD and type 3 diabetes, which represents a peculiar form of AD associated to glucose metabolism impairment. Indeed, epidemiological studies have shown that the consumption of dietary fiber, rich in ferulic acid, has been associated with a lower risk of developing AD along with diabetes [135,136].

These beneficial effects are likely ascribable to the well-known antioxidant and anti-inflammatory properties of this phenolic acid, as displayed in several preclinical studies. However, to date, only scanty clinical trials have been performed to confirm its therapeutic efficacy, most of the time involving foods and multicomponent food supplements containing ferulic acid rather than the pure substance [59]. This approach does not allow us to establish the exact dose of ferulic acid that could be effective to achieve specific clinical aims, for instance the amelioration of cognitive functions in AD subjects. Therefore, well-designed clinical trials with an appropriate number of subjects enrolled should be carried out in order to clarify the clinical efficacy of ferulic acid.

Regarding the potential application of ferulic acid against type 3 diabetes, the scientific research is still in its embryonic stage and needs to be encouraged. Indeed, to the best of our knowledge, only one in vivo study has investigated this approach, which deserves further attention due to the emergent evidence about the close relationship between AD and diabetes. More in vivo and mostly clinical trials should be performed in order to clarify the potential usefulness of ferulic acid in these age-related pathologies.

Another aspect which should be further investigated is the effect of ferulic acid in the modulation of microbiota, considering that it is now well known that functional foods could affect the brain via the gut-brain axis, and their intake can influence memory deficit [124]. Moreover, establishing the possible benefits of dietary sources of ferulic acid or herbal extracts in AD and type 3 diabetes also represents a future challenge.

In conclusion, the present narrative review highlights potential benefits of ferulic acid in AD and suggests a future interest in its use as a strategy to prevent and/or treat type 3 diabetes; however, more sound clinical evidence and clearer safety studies are needed to support a potential therapeutic application of ferulic acid in neurodegenerative diseases.

## Figures and Tables

**Table 1:** Ferulic acid (FA) amount in cereals, fruits, vegetables, and beverages.

	Source	mg of FA/100 g of Product	Reference
	Barley extract	1358–2293 ^a^	[31]
	Barley—whole grain flour	25–34 ^b^	[31]
	Corn—refined bran	2610–3300 ^a^	[31]
	Corn—dehulled kernels	174 ^a^	[31]
	Corn flour	38 ^a^	[31]
	Millet grits	26 ^a^	[26]
	Oat bran	33 ^a^	[31]
	Whole oat	25–35 ^a^	[31]
Cereals	Rice—endosperm cell wall	910 ^a^	[31]
	Rye bran	280 ^a^	[31]
	Rye—whole grain flour	86 ^a^	[31]
	Rye grain	90–117 ^b^	[27]
	Wheat—soft and hard bran	1351–1456 ^a^	[31]
	Wheat—fine bran	530–540 ^a^	[31]
	Wheat bran	300 ^a^	[26]
	Whole wheat flour	89 ^a^	[31]
	Barley—whole grain flour	25–34 ^b^	[31]
	Avocado	1.1 ^a^	[26]
	Broccoli	4.1 ^a^	[26]
	Carrot	1.5 ^a^	[26]
	Cauliflower	0.35 ^a^	[26]
	Eggplant	7.3–35 ^a^	[31]
	Garlic	0.63 ^a^	[26]
	Peanut	8.7 ^a^	[26]
	Red cabbage	6.3 ^a^	[26]
Vegetables and Fruits	Soybean	12 ^a^	[26]
	Spinach	7.4 ^a^	[31]
	Tomato	0.29–6 ^a^	[31]
	White cabbage	0.27 ^a^	[26]
	Banana	5.4 ^a^	[26]
	Blueberry fruits	290–1697 ^a^	[20]
	Grapefruit	11.6 ^a^	[26]
	Mandarin	9.24 ^a^	[26]
	Chokeberries	0.01–2.8 ^a^	[32]
	Orange	0.3 ^a^	[26]
	Plum	1.47 ^a^	[26]
	Strawberry	12.17 ^a^	[26]
	Apple juice	0.1 ^a^	[26]
	Beer	0.95 ^a^	[26]
Beverages	Black tea	0.16 ^a^	[26]
	Coffee	9.1 ^a^	[26]
	Orange juice	4.7 ^a^	[26]

^a^ mg/100 g of fresh edible fraction of foods; ^b^ mg/100 g of dry matter.

**Table 2:** Amount of ferulic acid (FA) in representative medicinal plants.

Species	Family Name	Source	mg of FA/g of Product	Reference
*Angelica sinensis* (Oliv.) Diels	Umbelliferae	roots	0.049–1.502 ^a^	[38]
*Angelica sinensis* (Oliv.) Diels	Umbelliferae	roots ^b^	0.018–0.035	[39]
*Ferula assa-foetida* L.	Umbelliferae	leaves ^c^	4.62	[35]
*Ferula assa-foetida* L.	Umbelliferae	gum ^c^	3.72	[35]
*Hyssopus officinalis* L.	Lamiaceae	herb	0.45 ^d^	[41]
*Lavandula angustifolia* Mill.	Lamiaceae	flowers ^b^	1.31	[42]
*Lavandula angustifolia* Mill.	Lamiaceae	flowers	0.1 ^d^	[41]
*Lavandula x intermedia* Emeric ex Loisel	Lamiaceae	flowers ^b^	0.11	[42]
*Mentha x piperita* L.	Lamiaceae	leaves ^b^	0.5	[42]
*Micromeria graeca* L.	Lamiaceae	aerial parts ^b^	0.27	[42]
*Micromeria Juliana* (L.) Benth. ex Rchb.	Lamiaceae	aerial parts ^b^	0.28	[42]
*Micromeria thymifolia* (Scop.) Fritsch	Lamiaceae	aerial parts ^b^	0.93	[42]
*Origanum majorana* L.	Lamiaceae	herb	0.05 ^d^	[41]
*Rosmarinus officinalis* L.	Lamiaceae	leaves	0.05 ^d^	[41]
*Salvia officinalis* L.	Lamiaceae	leaves ^b^	0.77	[42]
*Salvia* officinalis L.	Lamiaceae	leaves	0.05 ^d^	[41]
*Salvia miltiorrhiza* B.	Lamiaceae	roots ^e^	8.32 ^a^	[43]
*Teucrium arduini* L.	Lamiaceae	aerial parts ^b^	0.97	[42]
*Teucrium chamaedrys* L.	Lamiaceae	aerial parts ^b^	1.37	[42]
*Teucrium montanum* L.	Lamiaceae	aerial parts ^b^	1.70	[42]
*Teucrium polium* L.	Lamiaceae	aerial parts ^b^	1.38	[42]
*Thymus vulgaris* L.	Lamiaceae	aerial parts ^b^	0.49	[42]

^a^ µg/mL; ^b^ ethanolic extract; ^c^ hydroethanolic extract; ^d^ mg/g dry matter; ^e^ extract obtained from subcritical water extraction.

**Table 3:** Neuroprotective properties of ferulic acid in animal models of AD.

Study	Animal Models and Species (Number, Sex, Age)	Administration	Outcome		
			Behavioral Change	Neuropathological Change	Biochemical Change
Yan et al., 2001 [95]	i.c.v. injection of Aβ_1–42_ ICR mice (10, M, 4 weeks)	0.002%, 0.004%, and 0.006% (*w*/*v*) Free drinking 1, 2, 3, or 4 weeks	↑ memory	↓ Hippocampus GFAP and IL-1β immunoreactivities	↑ Cortex Acetylcholine level but not statistically significant
Kim et al., 2004 [96]	i.c.v. injection of Aβ_1–42_ ICR mice (6, M, 4 weeks)	0.006% (*w*/*v*) Free drinking 4 weeks	NA	↓ microglial activation	↓ IFN-γ
Cho et al., 2005 [97]	i.c.v. injection of Aβ_1–42_ ICR mice (6, M, 4 weeks)	0.006% (*w/v*) Free drinking 4 weeks	NA	↓ astrocytes activation	↓ hippocampal oxidative stress
Jin et al., 2005 [98]	i.c.v. injection of Aβ_25–35_ Sprague Dawley rats (7 weeks)	50, 100 or 250 mg/kg ig 3 weeks	NA	↓ astrocytes activation	↓ IL-1β and FasL ↓ p-p38 MAPK and caspase-3 ↑ ERK-1/2 and Akt/PKB activation
Jin et al., 2006 [99]	i.c.v. injection of Aβ_1–40_ Sprague Dawley rats (8 weeks)	100 or 200 mg/kg ig 3 weeks	NA	↑ hippocampal CA1 pyramidal neurons	↓ p-MKK3/MKK6, p-p-38 MAPK ↑ p-MAPKAPK-2, p-Hsp27, PARP ↓ IL-1β ↓ caspase-9, -3 and -7 activation
Kim et al., 2007 [100]	i.p. injection of TMT ICR mice (8, M)	0.002% or 0.005% (*w/v*) Free drinking 28 days	↓ memory impairment	NA	↑ ChAT activity
Mamiya et al., 2008 [101]	i.c.v. injection of BSO ICR mice (10/15, M, 25 weeks)	0.5, 1, or 5 mg/kg sc 6 days	↑ recognition memory ↑ short-term memory	↓ protein oxidation ↓ carbonyl protein levels in forebrains	NA
Jin et al., 2008 [102]	Aged Sprague Dawley rats (M, 21 months)	100 or 200 mg/kg in food 4 weeks	NA	↓ microglia and astrocytes activation in cortex and hippocampus Better arrangement of hippocampal CA1 pyramidal neurons	↓ IL-1β, p-MKK4, p-JNK, p-c-Jun ↑ p-ERK-1/2, p-MEK 1/2 ↓ caspase -3 and -7 activation
Hamaguchi et al., 2009 [103]	Mice double mutation K670N-M671L Tg2576 mice (10, F, 5 months)	0.5% in food 10 months	NA	↓ Aβ deposits	NA
Beibei et al., 2011 [104]	Injected KA into hippocampus CA1 region KM mice (10, M and F, 6 weeks)	20, 40 and 80 mg/kg ig 30 days	↑ learning and cognitive skills	↓ GFAP in hippocampal CA1 region	NA
Yan et al., 2013 [105]	APP/PS1 mice (5, F, 6 months)	5.3 and 16 mg/kg/d Free drinking 6 months	↑ memory	↓ Aβ_1–42_ and Aβ_1–40_ levels	↓ Il-1β
Mori et al., 2013 [106]	PSAPP C57BL/6J mice (12, M and F, 6 months)	30 mg/kg ig 6 months	↓ behavioral impairment	↓ Aβ deposits ↓ neuroinflammation and oxidative stress ↓ microglial and astroglial activation	↑ Iba1 ↓ TNF-a, IL-1β, Sod1, catalase, Gpx1 ↓ GFAP
Tsai et al., 2015 [107]	i.c.v. injection of Aβ_1–40_ SD rats (10–12, M, 9 weeks)	50 and 100 mg/kg ig 2 weeks	↓ cognitive function impairment ↑ improve memory	NA	↑ GSH, SOD ↓ Zn-SOD and AChE Activity
Kikugawa et al., 2016 [89]	i.c.v. injection of Aβ_25–35_ C57BL/6 J mice (6, M, 6 weeks)	0.1 µmol/g/day po 42 days	↑ contextual freezing response impairment	↑ neurons survival	NA
Mori et al., 2017 [108]	APP/PS1 C57BL/6J mice (8, M and F, 12 months)	30 mg/kg ig 3 months	↑ memory	↓ Aβ deposits ↓ astrocytosis, microgliosis, synaptotoxicity ↓ neuroinflammation and oxidative stress	↓ sAPP-α/holo-APP and β-oligomers ↑ ADAM10 and ↓ BACE1 ↑ synaptophysin ↓ TNF-α, IL-1β, SOD1, GPx1
Yue et al., 2017 [109]	APP/PS1 C57BL/6 mice (10, 5 weeks)	20, 40, 100 mg/kg ig 7 days	NA	↓ apoptosis and oxidative stress	↑ Bcl-2 and ↓ Bax, p-JNK, p-C-Jun, Caspase3 ↓ MDA and ↑ SOD
Rui et al., 2018 [110]	Injected KA into hippocampus CA1 region KM mice (M and F, 6 weeks)	20, 40, and 80 mg/kg 30 days	N/A	↓ positive GFAP cells in cerebral cortical glial cells	↓ IL-1β, IL-6, and TNF-α
Zafeer et al., 2019 [111]	ICV-STZ Wistar rats (6, M, 2 months)	100 mg/kg po 21 days	↓ spatial memory and learning loss	↓ oxidative stress ↓ mitochondrial damage	↓ ROS ↑ Drp-1, PGC1-α ↓ Mfn2, BAX, Cytochrome-C, LPO, and DNA fragmentation
Mori et al., 2019 [112]	APP/PS1 mice (8, M and F, 12 months)	30 mg/kg ig 3 months	↑ memory	↓ Aβ deposits ↓ astrocytosis and microgliosis ↓ neuroinflammation and oxidative stress ↓ synaptotoxicity	↓ amyloidogenic APP cleavage ↓ ADAM10 and BACE1 ↓ GFAP and Iba1 ↓ TNF-α and IL-1β, ↓ SOD1 and GPx1 ↑ synaptophysin
Qian et al., 2019 [113]	Injecting Aβ_1–42_ into the lateral ventricle KM mice (10, M, 6 weeks)	0.1 and 0.4 g/kg ig	↑ spatial positioning memory	↓ morphological changes ↓ Aβ generation	↓ Drp1, CnAα, CnAβ, and BACE1 ↓ Tau and pS396 protein
Wang et al., 2021 [114]	APP/PS1 mice (6 months)	20 mg/kg/day Free drinking 30 days	↑ spatial memory	↑ capillary density and diameter ↑ whole-brain blood vessels ↓ Aβ plaque deposits ↓ area of aggregative microglial cells ↓ ET1- mediated vasoconstriction and prevention of CBF reduction	↓ BACE1

↑, increase; ↓, decrease; NA, not applicable, as the parameter has not been determined; IFN-γ, interferon-γ; IL-1β, interleukin-1 β; FasL, Fas ligand; p-p38 MAPK, p38 mitogen-activated protein kinases; ERK-1/2, Extracellular signal-regulated kinases 1/2; Akt/PKB, protein kinase B; p-MKK3/MKK6, phosphorylated MAP kinases kinase 3/phospho-MAP kinases kinase 6; p-MAPKAPK-2, phosphorylated MAPK activating protein kinase 2; p-Hsp27, 27 kDa heat shock protein; PARP, poly(ADP-ribose) polymerase; ChAT, choline acetyltransferase; p-MKK4, phosphorylated MAP kinases kinase 4; p-JNK, phosphorylated c-Jun N-terminal Kinase; p-c-Jun, phosphorylated c-Jun; p-MEK 1/2, phosphorylated mitogen-activated protein kinase kinase 1/2; Iba1, Ionized calcium binding adaptor molecule 1; TNF-α, tumor necrosis factor α; SOD1, superoxide dismutase 1; Gpx1, glutathione peroxidase 1; GFAP, glial fibrillary acidic protein; GSH, reduced glutathione; AChE, acetylcholinesterase; sAPP-α/holo-APP, soluble amyloid β-protein precursor α/holo- amyloid β-protein precursor; ADAM10, a disintegrin and metalloproteinase domain-containing protein 10; BACE1, β-site APP-cleaving enzyme; Bcl-2, B-cell lymphoma 2; Bax, Bcl-2–associated protein x; MDA, malondialdehyde; IL-6, interleukin 6; ROS, reactive oxygen species; ET1 endothelin-1; LPO, lipid peroxidation; Drp-1, dynamin related protein-1; PGC1-α, peroxisome proliferator-activated receptor gamma coactivator 1-α; Mfn2, mitofusin 2; CnA-α, calcineurin A-α; CnA-β, calcineurin A-β; CBF cerebral blood flow; TMT trimethyltin chloride.

## Data Availability

Not applicable.
